# A scoring system to predict local progression-free survival in patients irradiated with 20 Gy in 5 fractions for malignant spinal cord compression

**DOI:** 10.1186/s13014-018-1203-y

**Published:** 2018-12-29

**Authors:** Dirk Rades, Antonio J. Conde-Moreno, Jon Cacicedo, Theo Veninga, Barbara Segedin, Karmen Stanic, Steven E. Schild

**Affiliations:** 1 Department of Radiation Oncology, University of Lübeck, Ratzeburger Allee 160, 23562 Lübeck, Germany; 2 Department of Radiation Oncology, University Hospital and Polytechnic La Fe, Valencia, Spain; 3 Department of Radiation Oncology, Cruces University Hospital, Barakaldo, Vizcaya Spain; 4 Department of Radiotherapy, Dr. Bernard Verbeeten Institute, Tilburg, Netherlands; 5 Department of Radiotherapy, Institute of Oncology Ljubljana, Ljubljana, Slovenia; 6 Department of Radiation Oncology, Mayo Clinic Scottsdale, Scottsdale, AZ USA

**Keywords:** Metastatic spinal cord compression, Radiotherapy alone, 20 Gy in 5 fractions, Local progression-free survival, Prognostic instrument

## Abstract

**Background:**

Local progression-free survival (LPFS = stable or improved motor function/resolution of paraplegia during RT without in-field recurrence following RT) is important when treating metastatic spinal cord compression (MSCC). An instrument to estimate LPFS was created to identify patients appropriately treated with short-course RT instead of longer-course RT plus/minus decompressive surgery.

**Methods:**

In 686 patients treated with 20 Gy in 5 fractions alone, ten characteristics were retrospectively analyzed for LPFS including age, interval between tumor diagnosis and RT of MSCC, visceral metastases, other bone metastases, primary tumor type, gender, time developing motor deficits, pre-RT gait function, number of vertebrae affected by MSCC, and performance score. Characteristics significantly (*p* < 0.05) associated with LPFS on multivariate analyses were incorporated in the scoring system. Six-month LPFS rates for significant characteristics were divided by 10, and corresponding points were added.

**Results:**

On multivariate analyses, visceral metastases (*p* < 0.001), tumor type (*p* = 0.009), time developing motor deficits (*p* < 0.001) and performance score (*p* = 0.009) were associated with LPFS and used for the scoring system. Scores for patients ranged between 24 and 35 points. Three groups were designed: 24–28 (A), 29–31 (B) and 32–35 (C) points. Six-month LPFS rates were 46, 69 and 92%, 12-month LPFS rates 46, 63 and 83%. Median survival times were 2 months (61% died within 2 months), 4 months and ≥ 11 months (median not reached).

**Conclusions:**

Most group A patients appeared sub-optimally treated with 20 Gy in 5 fractions. Patients with survival prognoses ≤2 months may be considered for best supportive care or single-fraction RT, those with prognoses ≥3 months for longer-course RT plus/minus upfront decompressive surgery. Many group B and most group C patients achieved long-time LPFS and appeared sufficiently treated with 20 Gy in 5 fractions. However, based on previous data, long-term survivors may benefit from longer-course RT.

## Background

In most countries worldwide, radiotherapy (RT) alone is the most common treatment for metastatic spinal cord compression (MSCC) [1, 2]. For MSCC, different dose-fractionation schedules are used, and of these, 30 Gy in 10 fractions over 2 weeks and 20 Gy in 5 fractions over 1 week are most commonly employed. Short-course RT with 20 Gy in 5 fractions or 8–10 Gy in 1 fraction has become quite popular to keep the treatment time as short as possible for this palliative situation, [3, 4]. This trend was supported by previous studies reporting this regimen was non-inferior to longer ones lasting 2 to 4 weeks with respect to motor function and post-trreatment ambulation rates [5–7]. One randomized trial, limited to patients with poor and intermediate survival prognoses, 20 Gy in 5 fractions was non-inferior to 30 Gy in 10 fractions with respect to local progression-free survival (LPFS) [7]. The question remains whether 20 Gy in 5 fractions is appropriate for patients with a favorable survival prognosis?

LPFS is a very important endpoint for the treatment of MSCC, because it takes into account the response of MSCC to RT and post-treatment local control of MSCC. Both deterioration of motor function during RT and symptomatic in-field recurrence of MSCC are disasterous for patients, since both situations generally result in severe pain, neurologic deficits and, if untreated, in complete paraplegia [1, 2]. Thus, LPFS achieved with 20 Gy in 5 fractions can be used as an indicator to judge whether a patient with MSCC is appropriately treated with this short-course regimen. To identify patients who may be good candidates for 20 Gy in 5 fractions rather than for longer-course programs plus/minus upfront decompressive surgery, it would be helpful for the treating physicians if they could use an instrument that estimates the LPFS prior to treatment. This study aimed to create this instrument in a large cohort of patients irradiated short-course RT alone for MSCC. Since a previous matched-pair study comparing 20 Gy in 5 fractions and 8 Gy in 1 fraction showed a trend in favor of 20 Gy in patients with intermediate survival prognoses and a significantly better local control of MSCC in patients with favorable prognoses, the present study was limited to patients who received 20 Gy in 5 fractions to avoid a selection bias caused by the dose-fractionation regimen [8].

.

## Methods

A total of 686 patients treated with 20 Gy in 5 fractions over 1 week without upfront decompressive surgery were included in this retrospective study. The present study was approved by the ethics committee of the University of Lübeck (reference number: 18-232A) and performed in accordance with the precepts established by the Helsinki Declaration. Criteria for inclusion in this study included motor deficits of the lower extremities due to MSCC, confirmation of MSCC by spinal magnetic resonance imaging (MRI) or computed tomography (CT), no other severe neurologic disease associated with motor weakness, presentation to a surgeon prior to RT, and corticosteroid treatment during RT. Radiotherapy was performed with 6–18 MV photon beams from a linear accelerator. Treatment volumes generally included one normal vertebra above and below those vertebrae involved. Ten pre-treatment characteristics were analyzed for a potential association with LPFS. These characteristics included age at the time of RT (≤65 vs. ≥66 years, median age: 66 years), interval between initial tumor diagnosis and RT of MSCC (≤15 vs. > 15 months, [9]), visceral metastases at the time of RT (no vs. yes), other bone metastases at the time of RT (no vs. yes), primary tumor type (breast cancer vs. prostate cancer, vs. myeloma vs. lung cancer vs. cancer of unknown primary vs. renal cell carcinoma vs. colorectal cancer vs. other tumors), gender, time developing motor deficits prior to RT (1–7 vs. 8–14 vs. > 14 days [5]), gait function prior to RT (not ambulatory vs. ambulatory), number of vertebrae affected by MSCC (1–2 vs. 3–4 vs. ≥5, [5]), and Eastern Cooperative Oncology Group (ECOG) performance score (1–2 vs. 3 vs. 4) (Table 1). In the entire cohort, no patient had an ECOG performance score of 0. Patients were assessed for motor function prior to RT, directly after RT, at 1 month following RT, and additionally if they developed progressive or new motor deficits during follow up. For assessment, the following 5-grade scale [10] was applied: 0 = normal strength; 1 = ambulatory without aid, 2 = ambulatory with aid, 3 = not ambulatory, 4 = complete paraplegia. Improvement and deterioration were counted when there was a change of at least one grade. In patients with progressive or new motor deficits, magnetic resonance imaging scans were obtained and reviewed by neuro-radiologists to differentiate between a recurrence of MSCC and other causes.

**Table 1:** Distribution of the investigated pre-treatment characteristics

	N patients’
	(%)
Age	
≤ 65 years	332 (48)
≥ 66 years	354 (52)
Interval between initial tumor diagnosis and RT of MSCC	
≤ 15 months	395 (58)
> 15 months	291 (42)
Visceral metastases at the time of RT	
No	354 (52)
Yes	332 (48)
Other bone metastases at the time of RT	
No	237 (35)
Yes	449 (65)
Type of primary tumor	
Breast cancer	134 (20)
Prostate cancer	140 (20)
Myeloma/lymphoma	56 (8)
Lung cancer	157 (23)
Unknown primary	69 (10)
Renal cell carcinoma	41 (6)
Colorectal cancer	24 (3)
Other tumors	65 (9)
Gender	
Female	265 (39)
Male	421 (61)
Time developing motor deficits prior to RT	
1–7 days	228 (33)
8–14 days	177 (26)
> 14 days	281 (41)
Gait function prior to RT	
Not ambulatory	281 (41)
Ambulatory	405 (59)
Number of vertebrae affected by MSCC	
1–2	279 (41)
3–4	260 (38)
≥ 5	147 (21)
ECOG performance score	
1–2	317 (46)
3	320 (47)
4	49 (7)

LPFS was defined as maintaining stable or improved motor function or, resolution of paraplegia during RT without clinical and radiologic evidence of an in-field recurrence of MSCC following RT. Time was referenced from the last day of RT. LPFS rates were calculated with the Kaplan-Meier-method, and differences between Kaplan-Meier curves of each investigated characteristic were compared with the log-rank test (univariate analyses) [11]. A difference was considered significant if the *p*-value ≤0.05. Those characteristics that were significant were included in multivariate analyses performed with the Cox proportional hazards model. If both ambulatory status and performance score were significant on univariate analysis, two separate multivariate analyses were performed, each including one of these two characteristics, as they are confounding variables. Also for multivariate analyses, *p*-values < 0.05 were considered significant. Those characteristics having significant associations with LPFS on both univariate and multivariate analyses were incorporated in the scoring system. For each patient, the 6-month LPFS rates of each significant characteristic (given in %) were divided by 10 to create a subscore for that characteristic. The subscores from the significant characteristics were summed to create a total score for various patient groups.

## Results

**Fig. 1:**
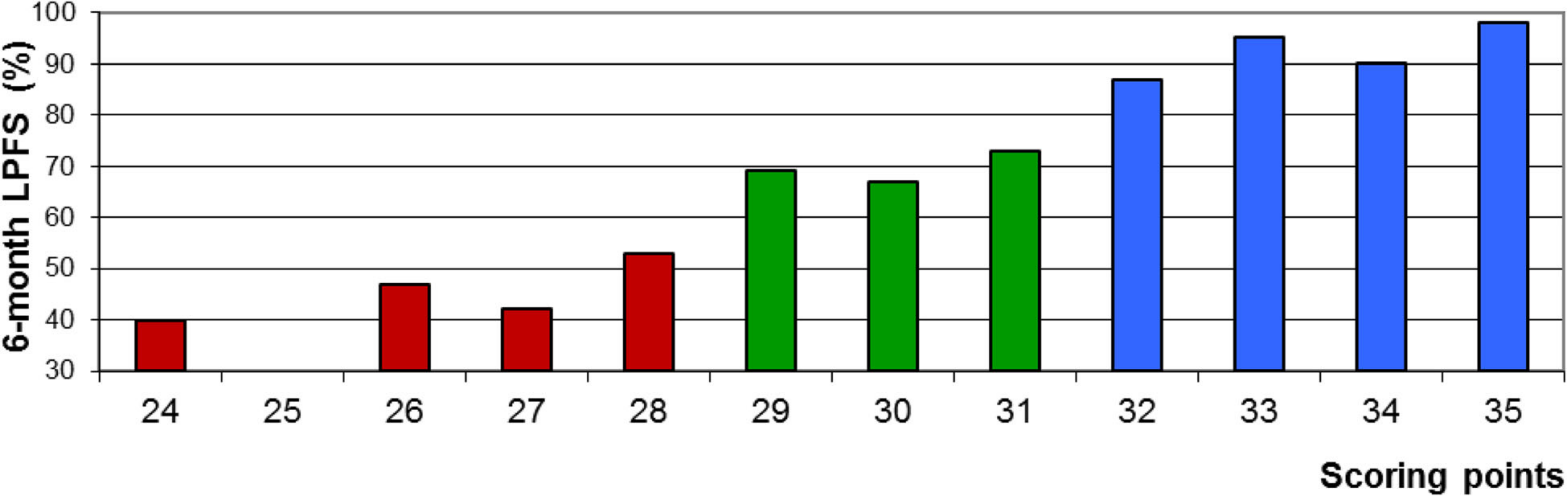
Scoring points for individual patients related to 6-month local progression-free survival (LPFS)

On univariate analyses of LPFS, improved outcome was significantly associated with an interval between initial tumor diagnosis and RT of MSCC of more than 15 months (*p* < 0.001), absence of visceral metastases (*p* < 0.001), absence of other bone metastases (*p* = 0.001), favorable primary tumor type (*p* < 0.001), slower development of motor deficits (> 14 days) prior to RT (*p* < 0.001), being ambulatory prior to RT (*p* < 0.001), affection of only 1–2 vertebrae by MSCC (*p* = 0.023) and ECOG performance score 1–2 (*p* < 0.001) (Table 2). On multivariate analyses, absence of visceral metastases (*p* < 0.001), favorable primary tumor type (*p* = 0.009), slower development of motor deficits prior to RT (*p* < 0.001) and ECOG performance score 1–2 (*p* = 0.009) maintained significance and were used to create the scoring system (Table 3). The scoring system was based on the 6-month LPFS rates of these four characteristics divided by 10. The corresponding scoring points for the characteristics are summarized in Table 4. After adding the scoring points of the four characteristics for each individual patient, the patient scores were obtained that ranged between 24 and 35 points (Fig. 1).

**Table 2:** Univariate analyses of local progression-free survival (LPFS)

	LPFS at 6 months	LPFS at 12 months	p-value
Age			
≤ 65 years	79	70	
≥ 66 years	74	69	0.23
Interval between initial tumor diagnosis and RT of MSCC			
≤ 15 months	71	66	
> 15 months	84	75	**< 0.001**
Visceral metastases at the time of RT			
No	83	78	
Yes	67	48	**< 0.001**
Other bone metastases at the time of RT			
No	85	77	
Yes	71	64	**0.001**
Type of primary tumor			
Breast cancer	88	80	
Prostate cancer	80	72	
Myeloma/lymphoma	93	89	
Lung cancer	68	61	
Unknown primary	66	66	
Renal cell carcinoma	77	51	
Colorectal cancer	41	41	
Other tumors	60	60	**< 0.001**
Gender			
Female	81	73	
Male	73	66	0.13
Time developing motor deficits prior to RT			
1–7 days	59	57	
8–14 days	81	75	
> 14 days	87	77	**< 0.001**
Gait function prior to RT			
Not ambulatory	70	63	
Ambulatory	81	74	**< 0.001**
Number of vertebrae affected by MSCC			
1–2	83	74	
3–4	75	69	
≥ 5	65	59	**0.023**
ECOG performance score			
1–2	87	78	
3	69	64	
4	55	55	**< 0.001**

Bold values = significant p-values

**Table 3:** Multivariate analyses of local progression-free survival (LPFS)

	Hazard ratio	95% confidence interval	p-value
Interval between initial tumor diagnosis and RT of MSCC (≤15 vs. > 15 months)	0.87	0.74–1.03	0.12
Visceral metastases at the time of RT (no vs. yes)	0.56	0.39–0.78	**< 0.001**
Other bone metastases at the time of RT (no vs. yes)	1.41	0.96–2.10	0.08
Type of primary tumor (breast cancer vs. prostate cancer vs. myeloma/lymphoma vs. lung cancer vs. unknown primary vs. renal cell carcinoma vs. colorectal cancer vs. other tumors)	1.91	1.17–3.09	**0.009**
Time developing motor deficits prior to RT (1–7 vs. 8–14 vs. > 14 days)	0.48	0.32–0.71	**< 0.001**
Gait function prior to RT (not ambulatory vs. ambulatory)	0.81	0.58–1.12	0.19
Number of vertebrae affected by MSCC (1–2 vs. 3–4 vs. ≥5)	1.28	0.82–1.98	0.27
ECOG performance score (1–2 vs. 3 vs. 4)	1.57	1.12–2.18	**0.009**

Bold values = significant *p*-values

**Table 4:** Scoring points of the four characteristics included in the scoring system

	LPFS at 6 months	p-value
Visceral metastases at the time of RT		
No	83	8
Yes	67	7
Type of primary tumor		
Breast cancer	88	9
Prostate cancer	80	8
Myeloma/lymphoma	93	9
Lung cancer	68	7
Unknown primary	66	7
Renal cell carcinoma	77	8
Colorectal cancer	41	4
Other tumors	60	6
Time developing motor deficits prior to RT		
1–7 days	59	6
8–14 days	81	8
> 14 days	87	9
ECOG performance score		
1–2	87	9
3	69	7
4	55	6

**Fig. 2:**
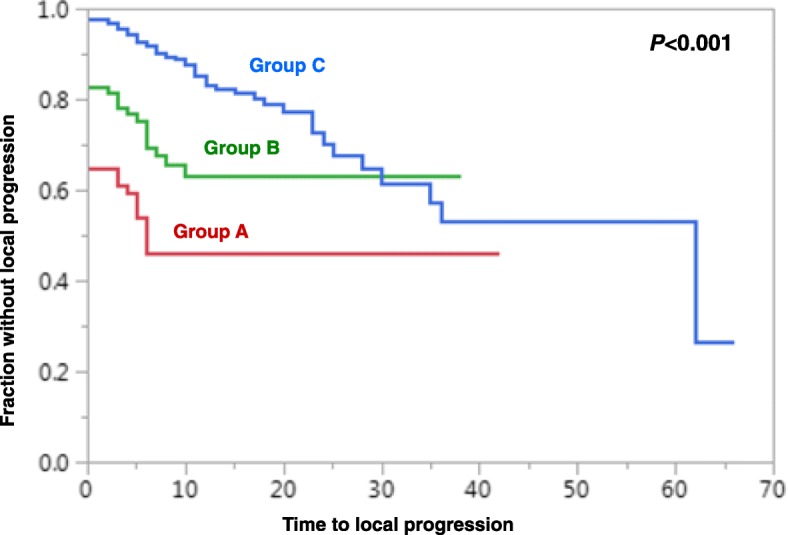
Comparison of the three prognostic groups A (24–28 points), B (29–31 points) and C (32–35 points) with respect to progression-free survival (univariate analysis)

**Fig. 3:**
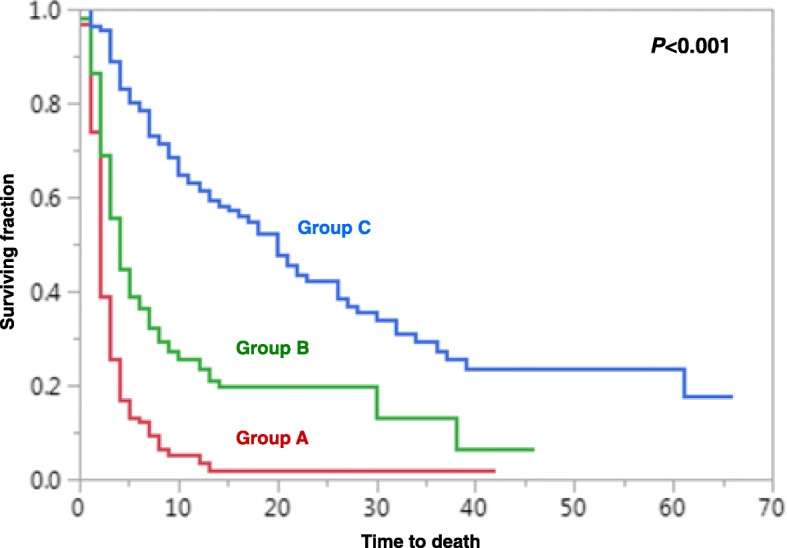
Comparison of the three prognostic groups A (24–28 points), B (29–31 points) and C (32–35 points) with respect to overall survival (univariate analysis)

Based on the 6-month LPFS rates of the patient scores (Fig. 1), the following three prognostic groups were segregated: 24–28 points (group A), 29–31 points (group B) and 32–35 points (group C). The LPFS rates were 46, 69 and 92% at 6 months following RT, and 46, 63 and 83% at 12 months following RT for groups A, B and C, respectively (Fig. 2). The survival rates of the three groups were 25, 56 and 88%, respectively, at 3 months and 12, 37 and 78% at 6 months (Fig. 3). Median survival times were 2 months, 4 months and ≥ 11 months (median not reached), respectively.

## Discussion

Since many patients with MSCC have a limited survival prognosis, radiation oncologists try to minimize the overall treatment time so patients don’t spend much of their remaining lives receiving radiotherapy [1, 2]. The most common short-course program used for MSCC is 20 Gy in 5 daily fractions administered on consecutive working days [2]. Generally, short-course programs are used for patients with a survival prognosis of a few months [3, 4, 7], whereas patients with a favorable prognosis, e.g. of more than 6 months, may benefit from longer-course programs with higher total doses in terms of improved local control of MSCC [12]. Thus, the dose-fractionation regimen used for an individual patient with MSCC is to a significant extent based on the patient’s estimated remaining lifespan.

Importantly, one randomized trial comparing upfront decompressive surgery plus 30 Gy in 10 fractions to 30 Gy in 10 fractions that demonstrated benefits in terms of survival and ambulatory function for the additional surgery included also patients with a very short survival time of only 3 months [13]. Therefore, upfront surgery appears to be a reasonable option not only for long-term survivors, and can be very helpful for patients who do not respond to or fail after RT. An in-field recurrence of MSCC may occur already a very few months following RT and is often associated with severe neurologic deficits and pain that significantly impair the patients’ quality of life [6, 9]. For many patients, longer-course RT plus/minus upfront decompressive surgery may be a better choice than short-course RT alone.

Thus, it appears important to identify prior to beginning short-course RT, those patients who would likely not respond sufficiently and those patients who are at a significant risk of developing an in-field recurrence of MSCC after a short-course regimen such as 20 Gy in 5 fractions. Both outcomes are included in the endpoint LPFS. Therefore, we aimed to create the first prognostic instrument that allows estimating the LPFS achieved with 20 Gy in 5 fractions alone and can aid physicians to decide whether a patient is a suitable candidate for this regimen. Based on four independent predictors of LPFS, three prognostic groups were designed with significantly different LPFS rates at 6 months following RT.

One of these four predictive factors was the time developing motor deficits prior to RT, which may lead to misunderstandings. In contrast to neurosurgical intervention, where an interval of < 48 h between onset of neurologic deficits and treatment is generally associated with more favorable treatment outcomes, a shorter interval between onset of the symptoms and the start of RT is associated with worse prognoses [5, 14–17]. The latter association can be explained by differences with respect to disruption of the local blood flow [14–16]. Faster development of MSCC can result in disruption of the arterial blood flow and consecutive ischemia and spinal cord infarction leading to irreversible motor deficits. In contrast, slower development of MSCC generally results in venous congestion and vasogenic edema of the white matter most often leading to reversible neurological deficits. These explanations were supported by a prospective study of patients receiving RT alone for MSCC [17]. Motor function improved in 86% of patients after a slow (> 14 days), in 29% after intermediate (8–14 days) and in 10% after fast (≤7 days) development of motor dysfunction (*p* < 0.001). Thus, time developing motor deficits prior to RT may be described as dynamics of the development of motor deficits.

In the present study, the 6-month LPFS rate in patients of group A (24–28 points) was only 46%, which was not optimal. Therefore, these patients should probably not be treated with 20 Gy in 5 fractions alone. The majority of these patients died within 3 months following RT and do, therefore, not meet the criteria of the Patchell study for upfront decompressive surgery [13]. Furthermore, longer-course RT is generally not considered appropriate for patients with such a short survival time, because in previous studies it did not result in higher rates of improvement of motor function and post-RT ambulatory status than short-course RT with 20 Gy in 5 fractions [5, 7]. Therefore, these patients may be considered for best supportive care alone or, in case of severe vertebral pain, for single-fraction RT [18]. One challenge for the treating physicians is the identification of those patients in group A, who likely will live 3 months or longer. These patients may be candidates for decompressive surgery followed by longer-course RT as in the Patchell trial or longer-course RT alone, depending on whether they also meet the other eligibility criteria of the Patchell trial, which included a Karnofsky performance score of ≥70, involvement of only one spinal segment by MSCC, MSCC not caused by a very radio-sensitive tumor (myeloma, lymphoma, germ cell tumors) and paraplegia lasting for not longer than 48 h [13]. In general, only a minority of patients with MSCC fulfil all of these criteria [1, 2]. Although not recommended for the treatment of MSCC in general, single-fraction stereotactic radiosurgery (SRS) or high-dose hypofractionated stereotactic body radiation therapy (SBRT), either alone or combined with less aggressive surgical approach of separation surgery, may be an option for carefully selected patients [19–21]. For estimation of a patient’s survival prognosis, a validated scoring system is available [22, 23]. The original scoring system (test group) was based on the data of 1852 patients irradiated for MSCC and included five prognostic groups. The 6-month survival rates were 4% (20–25 points), 11% (26–30 points), 48% (31–35 points), 87% (36–40 points) and 99% (41–45 points), respectively (*p* < 0.001) [22].

In the present study, the 6-month LPFS rate in patients of group B (29–31 points) was 69% and can be considered relatively favorable. Thus, most of these patients appeared appropriately treated with 20 Gy in 5 fractions. Those patients who are suboptimally treated with short-course RT must be identified as soon as possible. In case of inappropriate response to RT, decompressive surgery should be considered, particularly for those patients fulfilling the Patchell criteria incuding a survival prognosis of ≥3 months [13, 22, 23]. For highly selected patients of group B, SRS or SBRT plus/minus separation surgery may be considered [19–21].

Patients of group C had the most favorable LPFS rates and even achieved good long-term results with a LPFS rate of 83% at 12 months. Therefore, these patients can be considered appropriately treated with 20 Gy in 5 fractions alone. However, 20 Gy in 5 fractions may not be optimal for patients with a favorable survival prognosis, since in previous studies, short-course RT was associated with significantly higher rates of in-field recurrences than longer-course programs, and the risk of developing such a recurrence increases with lifetime [5, 6]. For identification of patients with more favorable survival prognoses, the previously presented survival score can be used [22, 23]. Those patients of group C with a very favorable survival prognosis of 12 months or longer, who represent a large proportion of this group, likely benefit even from longer-course RT with total doses higher than 30 Gy and doses per fraction less than 3 Gy. In a previous matched-pair study of 382 patients irradiated for MSCC, 2-year LPFS rates were 90% after 37.5 Gy in 15 fractions or 40 Gy in 20 fractions compared to 68% after 30 Gy in 10 fractions (*p* = 0.013) [12]. Combined treatment of decompressive surgery and longer-course RT may be offered to those 10–15% of patients with MSCC who fulfill the eligibility criteria of the Patchell trial [13]. Again, SRS or SBRT plus/minus separation surgery could be an option for selected patients [19–21].

In all three groups, those patients who appeared appropriately treated with 20 Gy in 5 fractions and have a poor survival prognosis of only a few months may be considered for single-fraction RT with 8 Gy or 10 Gy instead of 20 Gy in 5 fractions, because in previous studies including randomized trials single-fraction RT was not inferior to multi-fraction short-course RT for MSCC in patients with a poor estimated survival [3, 4, 8].

**Fig. 4:**
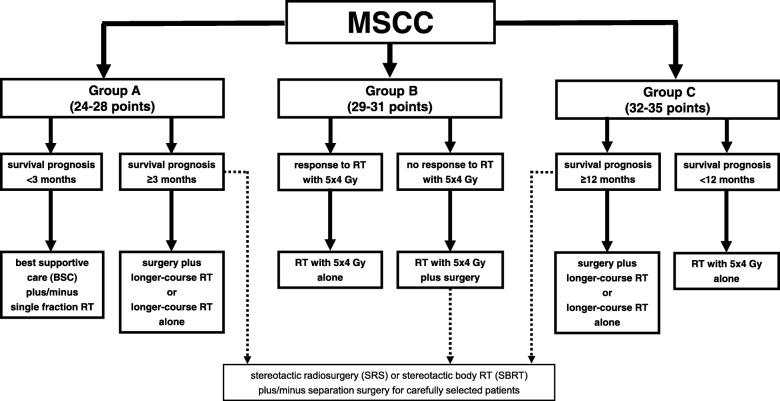
Treatment algorithm based on the results of the present study

The treatment recommendations stated above are summarized in an algorithm (Fig. 4). However, when following the given recommendations, one should bear in mind the retrospective design of this study. Retrospective studies always have a certain risk of including a hidden selection bias. Furthermore, the follow up of the patients was not performed at previously defined time points but only in case of new or progressive motor deficits indicating a possible in-field or out-field recurrence of MSCC. However, a randomized trial including an appropriately large cohort of patients to achieve an adequate statistical power will take several years and will not be available in a reasonable period of time. Therefore, a retrospective study performed in a very large cohort of patients is the best study design currently available.

## Conclusions

Most patients of group A appeared sub-optimally treated with 20 Gy in 5 fractions. Patients of these groups with survival prognoses of 2 months or less may be considered for best supportive care or 8 Gy in 1 fraction. Selected patients with a survival prognosis of 3 months or longer could benefit from longer-course RT plus/minus upfront decompressive surgery. Many patients of group B appeared appropriately treated with 20 Gy in 5 fractions. Those suboptimally treated with short-course RT must be identified a soon as possible, and decompressive surgery should be considered when applicable. Most patients of group C achieved long-time LPFS and appeared sufficiently treated with 20 Gy in 5 fractions. However, according to a previous study, long-term survivors could benefit from longer-course RT programs [12]. When following the given recommendations, the retrospective design of this study should be regarded. However, an appropriate randomized trial is not expected soon.

## A**bbreviations**

CT: Computed tomography
ECOG: Eastern Cooperative Oncology Group
LPFS: Local progression-free survival
MRI: Magnetic resonance imaging
MSCC: Metastatic spinal cord compression
RT: Radiotherapy
SBRT: Stereotactic body radiation therapy
SRS: Stereotactic radiosurgery
